# Patient Satisfaction with the Family Physician Program in Sabzevar, Iran

**DOI:** 10.5539/gjhs.v8n2p219

**Published:** 2015-06-25

**Authors:** Alireza Ghorbani, Pouran Raeissi, Ehsan Saffari, Nahid Reissi

**Affiliations:** 1 Department of Health Economics, School of Health Management and Information Sciences, Iran University of Medical Sciences, Tehran, Iran; 2 Department of Health Services Research, School of Health Management and Information Sciences, Iran University of Medical Sciences, Tehran, Iran; 3 Department of Epidemiology and Biostatistics, Sabzevar University of Medical Sciences, Sabzevar, Iran; 4 Department of Pediatrics, Hematology and Oncology, School of Medicine, Isfahan University of Medical Sciences, Isfahan, Iran

**Keywords:** patient satisfaction, family physician, patient preference, Iran

## Abstract

**Background and Objectives:**

Patient satisfaction with the family physician program is an important factor for more favorable treatment results. Evaluation of patient satisfaction improves the services and approximates them to patient’s preferences. The family physician program has been executed since late March, 2005 in Iran. This study aimed to measure patient satisfaction with family physician services and determines factors affecting the level of satisfaction in order to propose appropriate suggestions for providing medical services based on patients’ expectations.

**Methods:**

Forty-one centers provide healthcare services in rural and urban areas. The participants in this study comprised 1263 people. The data were collected by an inventory with 11 items about demographic specifications, waiting time and the importance of physician’s sex and 40 items for assessing the level of patient satisfaction.

**Results:**

A total of 1199 patients participated in the current study, 72.1% of them were female and 19.6% waited 10-20 minutes for receiving services. About 55.72% of the participants chose high and very high for the items of the inventory. Total satisfaction with the family physician program decreased with age (p-value= 0.029).Moreover, total satisfaction did not show any significant differences in different groups in terms of sex, place of residence, education level and marital status. Also family physicians’ sex did not affect patient satisfaction significantly. Based on results of regression model, an increase in patients’ age by one year decreased their satisfaction by 0.12 and level of satisfaction in rural patients was lower than that in urban patients by 7.93.

**Conclusions:**

The level of patient satisfaction with family physician services was moderate, which mostly arose from the components of the family physician program and services such as the waiting time, costs, welfare facilities, accessibility and the service-providing team rather than patients’ personal characteristics.

## 1. Introduction

Patient satisfaction is an essential indicator of the quality of health services (Ekram & Rahman, 2006; Fenton, Jerant, Bertakis, & Franks, 2012; Hjortdahl & Laerum, 1992; Lee, Tu, Chong, & Alter, 2008; Lemley & Marks, 2009; Mendoza, Smith, Eder, & Hickner, 2011) and the focus of decision makers in the health sector in the world (Hudak, Hogg-Johnson, Bombardier, McKeever, & Wright, 2004; Rundle-Thiele & Russell-Bennett, 2010). Patient satisfaction with family physician services is a multidimensional subject and reflects the expectations, values and experiences of patients (Devoe, Wallace, & Fryer, 2009; Kersnik, 2000; Lee et al., 2008; Virk, Kalia, Gupta, & Singh, 2013). Moreover, patient satisfaction is an important factor for higher adherence to medical instructions, higher loyalty to physicians, lower risk of complaint about the family physician and more favorable results of treatments (Fenton et al., 2012; Kersnik, 2000; Rundle-Thiele & Russell-Bennett, 2010; Sixma, Spreeuwenberg, & Vander Pasche, 1998; Wagner, Moseley, Grant, Gore, & Owens, 2002; Zgierska, rabago, & Miller, 2014). Patient satisfaction also indicates the technical and professional competence of family physicians (Hjortdahl & Laerum, 1992). Given that the family physician program should meet the credibility and forward-thinking to meet healthcare needs of the society (Organek et al., 2012), the evaluation of patient satisfaction allows the family physicians to obtain appropriate information about the level of fulfilled needs of patients and identify those dimensions of services with which the patients are less satisfied and make an effort to improve the services (Ekram& Rahman, 2006; Farzadi et al., 2011; Grogan, Conner, Willits, & Norman, 1995; Rundle-Thiele & Russell-Bennett, 2010; Zgierska et al., 2014).

According to Article 91 of the Fourth Economic, Social and Cultural Development Plan of Islamic Republic of Iran (2005-2009) stating that necessary measures should be performed to establish the health insurance and execute the family physician program and the referral system up to the end of the fourth development plan, the family physician program has been executed with the cooperation of the Ministry of Welfare and Social Security and the Ministry of Health and Medical Education in nomadic and rural areas and cities with a population fewer than 20,000 people since late March, 2005 (the onset of the Iranian year) (Khadivi, Golshiri, Farasat, & Khaledi, 2013; Torabian, Cheraghi, &Azarhomayoon, 2013). Considering a physician is not selected by people but by the Family Physician Program Management in healthcare center of each city, each physician covers 4,000 people in the program and people cannot choose their own family physician (Khadivi et al., 2013). Family physicians are general practitioners (Ministry of Health and Medical Education [MOHME], 2012) and a few of them have received training for the family physician (Bardella, 2009).

Various studies performed in different parts of the world, including Iran, reported different results about patient satisfaction with family physician services and factors influencing the satisfaction and stated that the level of satisfaction of most patients was related to components of services including access to healthcare, persistent availability of a physician’s services, costs, waiting time, information confidentiality, facilities of the waiting room and participation in medical decision-making rather than other factors such as the age, sex, income and occupation of patients (Alidoosti, Tavassoli, Delaram, Najimi, & Sharifirad, 2011; Farzadi et al., 2011; Gribben, 1993; Kersnik, 2000; Khadivi et al., 2013; Rahmqvist & Bara, 2010; Wetmore et al., 2014).

This study was conducted to measure patient satisfaction with family physician services and determines factors affecting the level of satisfaction in order to provide appropriate strategies and suggestions for supplying medical services based on patients’ expectations upon identification of the strengths and weaknesses of the program from their perspective.

## 2. Method

Supported by Sabzevar University of Medical Sciences in the northeast province of Iran, Khorasan-e Razavi, this study was performed in rural and urban areas with a population fewer than 20,000 people. The centers providing healthcare services in the mentioned areas included 35 rural healthcare centers and 6 urban healthcare centers. A general practitioner in each center worked as the family physician. The participants comprised 1263 people who were selected through stratified random sampling proportionate to the population covered in that center. In each center, the participants were selected through convenience sampling from the centers’ waiting lists. The objective of the study was explained for the selected patients who were over 15 years old and they were interviewed if they consented to participate in the study. The data related to the family physician’s months of service in each center and total months of service as a family physician were collected from the main office of the Family Physician Program Management.

The data were collected using an inventory adopted from the inventory for patient satisfaction assessment of the family physician, which was developed according to the state instruction for the family physician program and rural insurance (MOHME, 2012). The inventory consisted of 11 items about demographic specifications, waiting time, the importance of having a same sex physician and awareness of the place of paraclinic services and 40 items for assessing the level of patient satisfaction with the services provided by the family physician team over the previous year. The items of this part of the inventory were scored with a 5-point Likert scale (1 for never and 5 for very high). Minimum and maximum scores for participants’ total satisfaction were 40 and 200, respectively. The data were collected through interviewing with the patients and completing the inventory by trained interviewers over May and June in 2012. The Cronbach’s alpha of the inventory was 0.95 for the study population. The data were analyzed using the Wilcoxon rank-sum test, Kruskal-Wallis test and linear regression model. Statistical significance was set at p≤0.05.

### 2.1 Ethical Considerations

The Research Ethics Committee of Sabzevar University of Medical Sciences approved the study (license number 389091537). All health center managers were informed of the research intent prior to the study by the Research Office of Sabzevar University of Medical Sciences. All investigated subjects were also informed of the research objectives through the cover letter and were free to decide whether to complete the questionnaire. Anonymity was guaranteed for the respondents of the questionnaires.

## 3. Results

The study population comprised 1199 patients, of who 72.1%, 68.9% and 85.7% were females, housewives and married, respectively. Furthermore, 32.4% of the participants had primary school education and 86.4% were living in villages. Mean age of the participants was 41.4±16.2 years.

**Table 1:** Frequency of participants’ demographic specifications

Variable	Number	Percentage
**Sex**		
**Male**	335	27.9
**Female**	864	72.1
**Educational level**		
**Illiterate**	382	32.1
**Primary school**	387	32.3
**Middle school**	189	15.8
**Secondary school**	186	15.5
**Associate’s degree**	27	2.3
**Bachelor’s degree and higher**	16	1.3
**Seminary education**	4	0.3
**Unknown**	8	0.7
**Occupation**		
**Farmer**	200	16.7
**Worker**	38	3.2
**Employee**	19	1.6
**Housewife**	802	66.9
**Self-employed**	62	5.2
**Student**	33	2.8
**Unknown**	45	3.8
**Marital status**		
**Married**	1017	84.9
**Single**	166	13.8
**Widowed or divorced**	16	1.3
**Age**		
**Under 25 years**	177	14.8
**25-35 years**	318	26.5
**35-45 years**	254	21.2
**45-55 years**	162	13.5
**55-65 years**	113	9.4
**65-75 years**	72	6
**Over 75 years**	44	3.7
**Unknown**	59	4.9
**Importance of physician’s and patient’s being the same sex**		
**Very high**	239	19.9
**High**	214	17.8
**To some extent**	217	18.1
**Low**	92	7.7
**Not at all**	258	21.5
**Unknown**	179	14.9
**Waiting time for receiving the service**		
**Under 5 min**	94	7.8
**5-10 min**	246	20.5
**10-20 min**	235	19.6
**20-30 min**	225	18.8
**Over 20 min**	217	18.1
**Unknown**	182	15.2

The results of this study showed that the mean score of participants’ total satisfaction was 4.2±1.3, which indicated a moderate to high level of satisfaction with the services provided by the family physician program. Table 2 provides the frequency distribution of the answers given to each item of the inventory.

**Table 2:** Relative frequency distribution of patients’ responses to each item of the inventory

Item	Very low	Low	To some extent	High	Very high	No answer
**I trust in my physician’s secrecy.**	0.4	1.2	7.9	51.9	23.1	15.5
**I adhere to physician’s instructions and guidelines.**	0.5	0.9	10.1	49.1	24.6	14.8
**I trust in my physician’s performance.**	0.4	1.4	11.6	51.4	19.5	15.7
**Every time I should be referred to another physician, my physician refers me to a specialist.**	0.8	2.5	12.3	49.6	18.8	16
**The physician behaves me favorably.**	0.7	1.6	16	41.6	25.3	14.8
**The physician answers my questions and requests appropriately.**	0.7	2.2	15.7	48	18.3	15.1
**The nurse in the healthcare center has a good conduct.**	0.6	1.8	13.3	50	15.8	18.5
**The physician is present in the center at appointed hours.**	1.3	3.1	15.6	47.9	17.3	14.8
**I am satisfied with the timely presence of the physician for examinations.**	0.8	2.3	16.8	48.8	16.1	15.2
**Are you satisfied with the physician’s assistance for referring you to specialists?**	1.2	2.3	15.3	46.7	17.9	16.6
**I go to that center to receive health services again.**	1.7	2.3	12.4	49.5	14.5	19.6
**The physician spends enough time for examinations.**	1	3.2	17.3	46.7	16.7	15.1
**The services provided by the physician are suitable.**	0.8	2.5	18.7	47.7	14.6	15.7
**The healthcare center has a favorable heating system.**	2.3	5.6	15.2	46.5	15.6	14.8
**The healthcare center has suitable signposts.**	1.6	4.9	16.4	46.6	15.2	15.3
**The healthcare center has a favorable cooling system.**	1.8	5.2	17	45.7	15.4	14.9
**The place of providing service is suitable.**	1.2	3.1	18.8	45.6	15.3	16
**The physician diagnoses my disease properly.**	0.7	2.7	21.1	46.2	14.5	14.8
**I recommend other people to visit my family physician.**	2.3	3.3	15	45.6	14	19.8
**Are you satisfied with the pharmacy’s service provision?**	2.3	2.5	19.8	46.5	12.9	16
**I am aware of the services provided in this center.**	1.6	3.4	20.8	47.5	11	15.7
**There are enough chairs in the waiting room.**	2.8	7.9	16.1	42.4	15.5	15.3
**The therapeutic procedures of this center have been effective in my recovery.**	0.6	2.9	18.8	45.6	12.1	20
**The services provided to me match my needs.**	0.9	3.5	22.1	45.2	12.2	16.1
**The time the patient waits to be referred to a specialist is appropriate.**	1	3.3	19.6	45	10.3	20.8
**The services provided in this center are of favorable quality.**	0.8	2.1	21.7	45.2	10	20.2
**The waiting time for receiving the medication is suitable.**	0.2	2.2	14.8	42.1	12.3	28.4
**Are you satisfied with the premium?**	3.2	7.3	20.3	34.9	18.2	16.1
**The manner of follow-ups after being referred to a specialist is favorable.**	2.2	5.3	24.4	38.2	11.9	18
**The cost of services is suitable.**	4.6	7.8	23.1	35.4	13.9	15.2
**The number of medications in the pharmacy meets the patients’ needs.**	2.1	7.6	25.9	37.9	11	15.5
**The waiting time for receiving medical services is suitable.**	0.8	3.2	21.1	35.4	11.2	28.3
**The cost of medications is suitable.**	4.6	9.3	27.7	32	11.3	15.1
**Are you satisfied with laboratory services?**	2.8	5.1	21.1	34.1	8.2	28.7
**The diagnostic laboratory is favorably accessible.**	4.8	8.3	23	32.3	9.5	22.1
**The expenses of commuting to the healthcare center are suitable.**	9.8	13.3	23.4	26.8	10.3	16.4
**The waiting time for receiving services in emergencies is suitable.**	1.4	4.5	22.9	30.1	6.5	34.6
**The physician is easily accessible in emergencies.**	6	12.9	29.8	25.6	8.3	17.4
**The cost of laboratory and radiology services is suitable.**	6	10.8	25.4	21.9	5	30.9
**The waiting time for receiving radiology services is suitable.**	4.2	4.6	20.5	21.9	3.8	45

The results provided in Table 2 show that most of the participants were satisfied with different dimensions of the family physician program and physicians’ behavior. However, 20%-40% of them showed moderate to very low level of satisfaction with appropriateness of medical services, accessibility of the physician in emergencies, awareness of all types of services provided in these centers, appropriateness of the place where the services were provided, the availability of enough chairs in the waiting room, heating and cooling systems in the place where the services were provided, installation of suitable signposts in healthcare centers, cost-effectiveness of commuting to healthcare centers, proper diagnosis of the disease and the waiting time for receiving medications. However, they were dissatisfied mostly with the number of medications available in the pharmacies to meet their needs (35.6%), the cost of laboratory and radiology services (36.8%) and the cost of medications (41.6%) that indicated a considerable level of dissatisfaction (Table 2). The Kruskal-Wallis test was used to compare mean score of total satisfaction in terms of age, educational level and occupation (Table 3). Only the entirely answered questionnaires were included in the analysis because the total score of satisfaction could not be calculated for incomplete ones. The results showed a significant statistical difference in mean satisfaction of different age groups, as the mean total satisfaction with the family physician program decreased when the age increased, except the age group over 75 years in which the level of satisfaction had an upward trend. Although the mean total satisfaction with the family physician program increased with the educational level, the increase was not statistically significant.

**Table 3:** Comparison of mean total satisfaction in terms of patients’ age, educational level and occupation

Variable		Number	Mean ± standard deviation	P-value
**Age**	Under 25 years	56	94.61±16.01	0.029
	25-35 years	131	91.98±19.5	
	35-45 years	94	90.21±18.25	
	45-55 years	61	87.82±16.77	
	55-65 years	49	89.88±21.61	
	65-75 years	29	83.03±17.13	
	Over 75 years	19	90.45±18.63	
	
**Total**		439	90.45±18.63	

**Educational level**	Illiterate	152	88.61±18.03	0.397
	Primary school	147	88.99±19.22	
	Middle school	79	92.05±18.3	
	Secondary school	65	93.42±18.66	
	Associate’s degree	8	93.42±18.66	
	Bachelor’s degree and higher	2	100.5±24.75	
	Seminary education	1	99	
	
**Total**		454	90.2±18.59

Occupation	Farmer	85	89.91±18.64	0.621
	Worker	14	89±13.31	
	Employee	5	78.6±12.6	
	Housewife	296	90.96±18.35	
	Self-employed	28	90.07±23.34	
	Student	12	91.67±23.3	
	
**Total**		440	90.52±18.67

The Wilcoxon rank-sum test was used to measure the effect of sex, place of residence and marital status on the level of total satisfaction in the studied participants and the results did not show any significant difference between different groups.

**Table 4:** Comparison of mean total satisfaction in terms of patients’ sex, marital status and place of residence

Variable		Number	Mean ± standard deviation	P-value
Sex	Male	141	89.44±19.26	0.457
	Female	312	90.53±18.33	
Marital status	Single	61	88.82±17.57	0.545
	Married	386	90.66±18.84	
Place of residence	City	29	97.76±20.36	0.162
	Village	418	89.83±18.45	

The effect of family physicians’ characteristics including sex, months of service in the family physician program and months of service in the current healthcare center were studied as independent variables on the satisfaction with the family physician program as the dependent variable. In total, 60% of the participants (719 patients) had visited a male family physician. Months of service as a physician in the family physician program and in the current healthcare center supplying family physician services were 31.99±19.09 months and 24.98±16.55 months, respectively. Male and female participants’ satisfaction with female physicians (92.22±19.56 & 91.65±19.19, respectively) was higher than that with male physicians (88.30±19.11 & 89.82±17.78, respectively) although Pearson’s correlation coefficient showed no significant difference between male (P-value=0.274) and female (P-value=0.391) participants in terms of satisfaction with male and female physicians (four possible combinations of patients’ sex and physicians’ sex). In other words, family physicians’ sex did not affect patient satisfaction significantly. Moreover, Pearson’s correlation coefficient test showed that the level of male participants’ satisfaction with family physician services directly correlated with physician’s total months of service in the family physician program (r=0.01) and physician’s months of service in the current center (r=0.04) and the level of female participants’ satisfaction with the family physician services inversely correlated with physician’s total months of service in the family physician program (r=-0.025) and physician’s months of service in the current center (r=-0.054). However, the above correlations were not significant (P-value > 0.05).

The linear regression model was used to determine the effect of each variable on the level of satisfaction in patients visiting family physicians. Independent variables included sex, marital status, educational level, age, place of residence, physician’s sex, months of service and similarity of physician and patient in sex. According to the results of the regression model on age, an increase in patients’ age by one year decreased their satisfaction by 0.12. On the place of residence, the level of satisfaction in rural patients was lower than that in urban patients by 7.93.

**Table 5:** Results of the simple linear regression model

Variable	Coefficient	P-value
**Sex**	1.09	0.564
**Marital status**	1.84	0.475
**Educational level**	1.69	0.067
**Place of residence**	-7.93	0.027
**Distance to the center**	0.11	0.314
**Physician’s sex**	-2.58	0.155
**Physician’s months of service**	-0.02	0.743
**Physician’s months of service in the center**	-0.05	0.416
**Age**	-0.12	0.027
**Physician’s and patient’s being the same sex**	0.11	0.95

## 4. Discussion

It seems that the level of satisfaction with the family physician program in the studied area was moderate because 55.72% of the participants chose high and very high for the items of the inventory and 6.59% of them chose low and very low. This level of satisfaction indicated that the obligation of visiting a specific family physician assigned by the main office of the Family Physician Program Management for the population living in a geographical location (the rural healthcare center) reduced access to healthcare services consistent with patients’ expectations and influenced their trust in the accuracy of medical diagnoses and decisions. Patients are not allowed to change their family physician. Furthermore, patients might be satisfied with a family physician, who would be transferred to another geographical region following the decision of the main office of the Family Physician Program Management. Another point was that family physicians were paid per capita (MOHME, 2012) and thus, the patients’ level of satisfaction did not affect family physicians’ salary and physicians might not be motivated to be aware of patients’ level of satisfaction, provide high-quality services and try to increase patient satisfaction.

The maximum level of dissatisfaction pertained to commuting expenses to the healthcare center, as 9.8% of them chose very low for the relevant item. According to the executive instruction of the program, family physicians should go to all villages they cover, visit and treat patients (MOHME, 2012) in order that the expenses of transportation would be paid by the service providers not by the patients besides increasing the accessibility to heath-treatment services. Moreover, the commuting expenses might sound heavy for patients because they do not pay for the premium and 70% of the pay for visits and medications (MOHME, 2012); therefore, commuting expenses are sometimes several times as those of the medications and treatment.

Patient dissatisfaction with the cost of laboratory and radiology services was considerable. Radiology services were provided only at the second level of delivery services and laboratory services were provided in a few centers (12 centers). In this respect, patients should pay indirect expenses besides the expenses paid directly for the services. Furthermore, patients should pay the entire cost of services in some cases where the documents needed for referring them to higher levels were incomplete and the insurance organization did not pay for paraclinic services. The participants declared rather high dissatisfaction with the cost of medications, which was not unexpected, as they should pay 30% franchises.

They were also highly dissatisfied with the number of medications in pharmacies. Based on the executive instruction of the family physician program, 270 types of medication should be available in the pharmacy of the healthcare centers. However, the important point is that family physicians should not prescribe over 2.5 types of medications in each prescription; otherwise, they would be subjected to deductions. This limitation can affect patient satisfaction with accessibility to their desired number of medications.

The long waiting time for receiving services seemed normal to some extent regarding the high volume of demands for medical services and subsequently medicinal services in the centers. The reason is that, economically speaking, the decrease in cost of services after the development of insurance coverage increases the unnecessary demands that lead to long waiting lists besides the waste of resources and deprivation of people in need of services.

On the access to the family physician in emergencies, patients covered by the family physician program can use the family physician services once a week and only when they go to the healthcare centers personally or after making an appointment in the Health House and they rarely gain access to family physician from 4:00 pm to 7:30 am. Family physicians are not accessible on holidays or by telephone, and telephone is not a means of patient-family physician communication.

Moreover, the participants were not much satisfied with the facilities in the centers supplying family physician services because the rural insurance and family physician program were executed without any considerable changes in infrastructures of the rural healthcare centers and health houses in 2005, and the significant increase in the number of patients covered by rural insurance and their expectation for minimum facilities in healthcare centers and health houses had not received the attention of the program’s executors. Furthermore, rural health houses did not have enough space and a part of patients’ dissatisfaction was associated with limitations in the health houses.

Maximum level of participants’ satisfaction pertained to physicians’ secrecy, as 75% of the participants chose high and very high for the relevant item. Considering that the respect for patients’ rights was of special importance (Jafari, 2014) and confidentiality was of professional principles of medicine, family physicians could act favorably in this regard although they were working in small areas.

As mentioned before, the level of patient satisfaction with family physician services was moderate, which mostly arose from the components of the family physician program and services such as the waiting time, costs, welfare facilities, accessibility and the service-providing team rather than the patients’ personal characteristics although satisfaction is an emotional state under the influence of patients’ priorities and expectations (Baker, 1997).

After almost a decade of executing family physician program in Islamic Republic of Iran’s rural and urban areas with fewer than 20,000 people, the services provided in this new program were far from patients’ expectations. Therefore, with regard to the results of this study and to increase patient satisfaction with family physician services, especially in the first level of service provision, the state policy-making system can provide family physician services within people’s expectations and quality standards of services besides developing a purposeful system through redefining family physician and his role in providing healthcare to the people covered by the program, emphasizing the influence of family physicians on screening and preventing the diseases, facilitating patient-family physician communication, developing the scope of family physician services through expanding the 24-hour medical centers, informing the public about the objectives of the family physician program, culturalizing the proper use of medical and medicinal services, giving patients the chance of choosing and changing their family physician, using hybrid methods to supplement the services of family physician and healthcare team emphasizing the level of patient satisfaction, defining the standard service package qualitatively and quantitatively for higher accessibility to the services, determining the welfare standards of centers supplying the family physician services, training professional family physicians and promoting the referral system at the three levels of providing service.

Future studies are recommended to examine the relationship of patient satisfaction with the type and severity of the disease, physicians’ emotional intelligence, the regular presence of family physicians in villages covered by the program, types of services and mean number of drugs prescribed by physicians.

## 5. Conclusion

According to the results of this study, patient satisfaction with family physician services was at a moderate level and regarding that the family physician program has been executed in the Islamic Republic of Iran for fewer than 10 years, patient satisfaction can be significantly improved through taking small steps from revising the cost of services to promoting welfare facilities.

### 5.1 Ethical Considerations of the Study

Patients voluntarily participated in the study and were interviewed upon obtaining their informed consent. Moreover, the information they provided was completely confidential and recorded anonymously.

### 5.2 Limitations of the Study

The researchers had to use interview to collect the data because they predicted that a large number of participants were illiterate or of low literacy. However, the researchers tried to minimize the influence of interviewers on patients’ expression of opinions through holding three sessions of training. Moreover, women comprised a major part of the study sample because the study was performed at the end of the spring when most of the men were working on farms, and thus, the results of this study reflects men’s opinions less than that of women.
